# Leptin and environmental temperature as determinants of bone marrow adiposity in female mice

**DOI:** 10.3389/fendo.2022.959743

**Published:** 2022-10-06

**Authors:** Russell T. Turner, Kira L. Nesser, Kenneth A. Philbrick, Carmen P. Wong, Dawn A. Olson, Adam J. Branscum, Urszula T. Iwaniec

**Affiliations:** ^1^ Skeletal Biology Laboratory, School of Biological and Population Health Sciences, Oregon State University, Corvallis, OR, United States; ^2^ Center for Healthy Aging Research, Oregon State University, Corvallis, OR, United States; ^3^ Biostatistics Program, School of Biological and Population Health Sciences, Oregon State University, Corvallis, OR, United States

**Keywords:** leptin, BMAT, thermoneutral, room temperature, marrow adipose tissue, histomorphometry

## Abstract

Bone marrow adipose tissue (BMAT) levels are higher in distal femur metaphysis of female mice housed at thermoneutral (32°C) than in mice housed at 22°C, as are abdominal white adipose tissue (WAT) mass, and serum leptin levels. We performed two experiments to explore the role of increased leptin in temperature-enhanced accrual of BMAT. First, we supplemented 6-week-old female C57BL/6J (B6) mice with leptin for 2 weeks at 10 µg/d using a subcutaneously implanted osmotic pump. Controls consisted of *ad libitum* (*ad lib*) fed mice and mice pair fed to match food intake of leptin-supplemented mice. The mice were maintained at 32°C for the duration of treatment. At necropsy, serum leptin in leptin-supplemented mice did not differ from *ad lib* mice, suggesting suppression of endogenous leptin production. In support, *Ucp1* expression in BAT, percent body fat, and abdominal WAT mass were lower in leptin-supplemented mice. Leptin-supplemented mice also had lower BMAT and higher bone formation in distal femur metaphysis compared to the *ad lib* group, changes not replicated by pair-feeding. In the second experiment, BMAT response was evaluated in 6-week-old female B6 wild type (WT), leptin-deficient *ob/ob* and leptin-treated (0.3 μg/d) *ob/ob* mice housed at 32°C for the 2-week duration of the treatment. Compared to mice sacrificed at baseline (22°C), BMAT increased in *ob/ob* mice as well as WT mice, indicating a leptin independent response to increased temperature. However, infusion of *ob/ob* mice with leptin, at a dose rate having negligible effects on either energy metabolism or serum leptin levels, attenuated the increase in BMAT. In summary, increased housing temperature and increased leptin have independent but opposing effects on BMAT in mice.

## Introduction

Bone marrow adipose tissue (BMAT) is a fat depot spatially confined within the skeleton. The resident adipocytes are derived primarily from bone marrow mesenchymal stem cells. As reviewed (1–4), BMAT differs from other adipose depots in that the density and spatial distribution of adipocytes can vary dramatically within and among bones and adipocytes are interspersed among many other cell populations, including hematopoietic lineage cells. The function and regulation of BMAT is incompletely understood but has received considerable recent attention, particularly in regard to its role as a potential regulator of bone metabolism (5, 6). In rodents, conditions such as diet-induced obesity, chronic heavy alcohol consumption, severe caloric restriction and microgravity have been reported to result in increased BMAT. Notably, the same conditions are often associated with decreased osteoblast differentiation and/or activity, and bone loss (7–10). Mechanistically, some investigators have hypothesized that differentiation to adipocytes at the expense of osteoblasts and/or production of adipocyte-derived adipokines lead to a negative bone turnover balance (11, 12). However, a negative relationship between BMAT levels and osteoblasts is far from universal (6, 13–15) and, when the relationship occurs, causality has not been established. In total, the experimental evidence to date does not strongly support a deterministic model where reducing BMAT will invariably lead to increased bone mass (1).

Mice are commonly used as a mammalian model organism, in part because of their diminutive size, relatively short lifespan and ease of genetic manipulation. However, skeletal adaptations do not scale linearly with body size (16). In addition to being physically small, mice are daily facultative heterotherms and have a thermoneutral zone that is much higher than in humans (17). One outcome of this difference in thermoregulation is that mice housed at room temperature (~22°C) experience cold stress. Adaptation to chronic cold stress is recognized to have profound physiological effects that influence disease processes (18, 19). In contrast to larger rodents and humans, male and female mice lose cancellous bone in femur and lumbar vertebra well before growth has ceased (20). We have shown that cold stress induced by room temperature housing contributes to, and may be fully responsible for, premature cancellous bone loss in mice. Additionally, cold stress in growing mice results in compartment-specific reductions in bone accrual, reduced leptin levels, and decreased accrual of BMAT (21, 22).

There is strong circumstantial evidence that the adipokine leptin is an important negative regulator of BMAT in mice (23). Compared to C57BL/6J (B6) wild type (WT) mice, bone growth and turnover are lower and BMAT higher in long bones of leptin-deficient ob/ob mice (24–28). Furthermore, treatment of ob/ob mice with leptin by either intracerebroventricular or subcutaneous delivery normalizes bone growth and turnover and reduces BMAT levels (23, 25, 29, 30). On the other hand, there are circumstances where elevated leptin is associated with increased BMAT. For example, compared to mice housed at 22°C, housing B6 female mice at 32°C results in increases in serum leptin and BMAT (31). Additionally, high blood leptin levels in obese rodents are associated with increased BMAT (32). These discrepant findings, where higher levels of leptin can be associated with either increases or decreases in BMAT, suggest that environmental temperature has actions on BMAT that do not require leptin.

To better understand the role of leptin as a potential mediator of the response of BMAT to changes in environmental temperature, we performed 2 experiments where we measured BMAT 2 weeks following transfer of mice housed at room temperature (22°C) to near thermoneutral (32°C) housing. It is challenging to house mice at true thermoneutral because the thermoneutral point differs by several degrees between the dark and light photo periods (33). However, a constant temperature of 30-32°C approximates the midrange of thermoneutrality and was shown to prevent cold stress-induced cancellous bone loss in male and female mice as well as result in increased BMAT in female mice (21).

In the first experiment, we supplemented 6-week-old female B6 WT mice with leptin, administered for 2 weeks using a subcutaneously implanted osmotic pump, at 10 µg/d. Controls consisted of ad libitum (ad lib) fed mice and mice pair fed to match food consumption of leptin-supplemented mice. In the second experiment, BMAT response was evaluated in 6-week-old female B6 WT, leptin-deficient ob/ob and leptin-treated (0.3 μg/d) ob/ob mice housed at 32°C for the 2-week duration of treatment.

## Materials and methods

The experimental protocols were approved by the Institutional Animal Care and Use Committee at Oregon State University in accordance with the NIH Guide for the Care and Use of Laboratory Animals.

### Experiment 1: Effects of leptin supplementation on food intake, body composition, bone and bone marrow adiposity in female B6 mice

**Figure 1:**
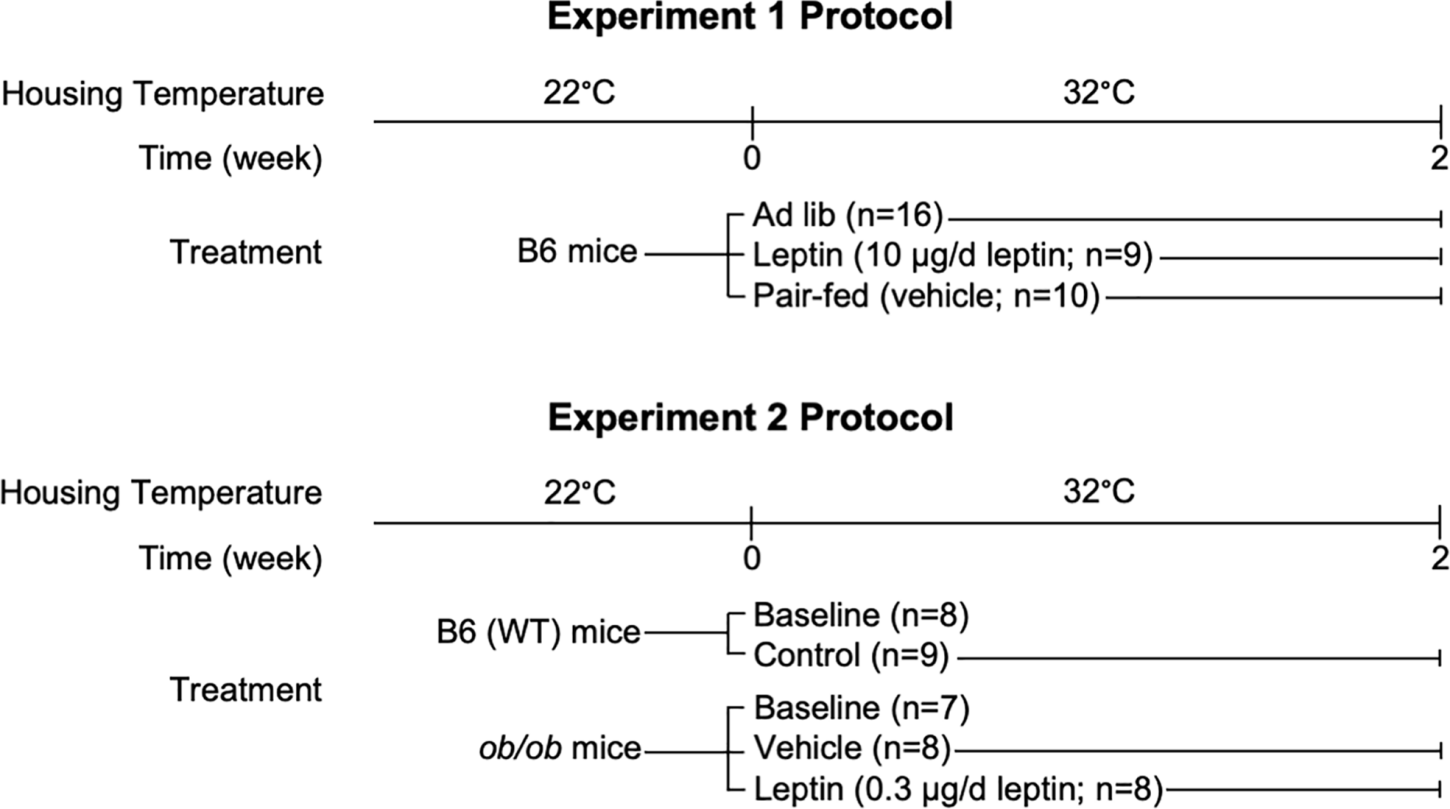
Experimental design.

Six-week-old female B6 mice were purchased from Jackson Laboratory (Bar Harbor, ME, USA). We chose 6-week-old mice because this age corresponds to near peak cancellous bone volume fraction in femur in the B6 female (20). The animals were placed at 32°C upon arrival and maintained individually housed on a 12 h light:12 h dark cycle. Near thermoneutral housing was used to prevent cold stress associated with subthermoneutral housing (e.g., standard room temperature, ~22°C) (31, 34). The mice were randomized by weight into one of 3 treatment groups: (1) ad lib (n=16), (2) leptin-supplemented (leptin) (n=9), or (3) vehicle-treated and pair-fed to leptin (pair-fed) (n=10) (**Figure 1**). Mouse leptin (10 µg/d, equivalent to 400 ng/h; 498-OB-05M, R&D Systems, Minneapolis, MN) or vehicle (20 mM Tris-HCL, Invitrogen, Carlsbad, CA) were delivered via subcutaneously implanted osmotic pumps (Alzet Model 1002, Durect Corporation, Cupertino, CA) to leptin and pair-fed mice, respectively as described (35). Food (Teklad 8604, Harlen Laboratories, Indianapolis, IN) and water were provided ad libitum to the ad lib and leptin-supplemented mice. The pair-fed mice were calorically restricted to match food intake of the leptin-supplemented mice. These mice also served as vehicle-treated surgery controls. We did not include B6 mice housed at 22°C as an additional control because a pilot study (data not shown) revealed no change in BMAT. Food intake was recorded daily for the 2-week duration of treatment. Body weight was recorded 2 days prior to implant surgery, on day of implant surgery (only mice undergoing surgery were weighed), and on days 4, 11, and 14 (necropsy) post-surgery. Calcein was administered at 4 and 1 d prior to necropsy to label mineralizing bone. Mice were fasted overnight and dual energy x-ray absorptiometry (DXA) was performed prior to necropsy. For tissue collection death was induced by cardiac exsanguination. Abdominal white adipose tissue (WAT) – perigonadal, mesenteric, perirenal, retroperitoneal – was excised and weighed. Samples of perigonadal WAT were stored in RNAlater for evaluation of gene expression. Interscapular brown adipose tissue (BAT) was also excised and stored in RNAlater for evaluation of gene expression. Femora were removed, fixed for 24h in 10% buffered formalin and stored in 70% ethanol for histomorphometric analysis. Tibiae were frozen in liquid nitrogen and stored at −80°C for analysis of gene expression.

### Experiment 2: Effects of leptin on body composition and bone marrow adiposity in leptin-deficient ob/ob female mice

Six-week-old female B6 WT mice and ob/ob mice on a B6 background were purchased from Jackson Laboratory (Bar Harbor, ME, USA). Following arrival, the B6 WT mice were randomized by weight into one of 2 groups: (1) baseline (n=8) and (2) no treatment control (n=9) (**Figure 1**). The ob/ob mice were randomized by weight into 3 groups: (1) baseline (n=7), (2) vehicle (n=8), or (3) leptin-treated (n=8). The baseline groups were sacrificed at the start of the experiment and treatment mice placed at 32°C. Mouse leptin (0.3 µg/d, equivalent to 12 ng/h; 498-OB-05M, R&D Systems, Minneapolis, MN) or vehicle (20 mM Tris-HCL, Invitrogen, Carlsbad, CA) were delivered via subcutaneously implanted osmotic pumps (Alzet Model 1002, Durect Corporation, Cupertino, CA) to leptin and vehicle mice, respectively. Infusion of leptin at 0.3 µg/d was shown to increase longitudinal bone growth and bone formation with minimal effects on hypothalamic gene expression in ob/ob mice (35). Food (Teklad 8604, Harlen Laboratories, Indianapolis, IN) and water were provided ad libitum to all groups. We did not include mice housed at 22°C as an additional control because a pilot study (data not shown) did not reveal a change in BMAT. As in Experiment 1, percent body fat (DXA) and abdominal WAT mass (perigonadal, mesenteric, perirenal, retroperitoneal) were recorded at necropsy. In addition, interscapular BAT was removed and stored in RNA later for evaluation of gene expression and femora were removed, fixed for 24h in 10% buffered formalin, and stored in 70% ethanol for histomorphometric analysis of bone marrow adiposity.

### Dual energy absorptiometry

Total body bone mineral content (g), bone area (cm^2^), bone mineral density (g/cm^2^) and percent body fat were determined using DXA (PIXImus2, Lunar, Madison, WI) under isoflurane anesthesia immediately prior to necropsy.

### Blood measurements

Serum leptin was measured using Mouse Leptin Quantikine ELISA Kit (R&D Systems, Minneapolis, MN), serum osteocalcin was measured using Mouse Gla-Osteocalcin High Sensitive EIA Kit (Clontech, Mountain View, CA), and serum Mouse C terminal telopeptides of type I collagen (CTX-1) was measured using ELISA kit (Life Sciences Advanced Technologies, St. Petersburg, FL) according to the respective manufacturer’s protocol. Intra-assay coefficient of variation (CV) for all ELISA assays were within the manufactures’ reported CVs of ≤ 5%.

### Histomorphometry

The methods used to measure static and dynamic bone histomorphometry have been described, with modifications for mice (36). In brief, distal femora were dehydrated in a graded series of ethanol and xylene, and embedded undecalcified in modified methyl methacrylate. Longitudinal sections (4 µm thick) were cut with a vertical bed microtome (Leica 2065) and affixed to slides precoated with 1% gelatin solution. One section/animal was mounted unstained for measurement of fluorochrome labels and marrow adipocytes using ultraviolet illumination. All data were collected using the OsteoMeasure System (OsteoMetrics, Inc., Atlanta, GA) in the distal femur metaphysis (10x) and distal femur diaphysis (6.7x) (**Supplemental Figure 1**). The sampling site for the distal femoral metaphysis was located 0.25 – 1.1 mm proximal to the growth plate, averaged 0.92 mm^2^ in area, and excluded primary spongiosa and cortical bone. The sampling site for the distal femoral diaphysis was located 2.0 – 2.7 mm proximal to the growth plate, averaged 0.63 mm^2^ in area, and excluded cortical bone. Proper depth was identified by a characteristic v-shaped growth plate and parallel diaphyseal cortices approximately equal in width (**Supplemental Figure 1**).

Fluorochrome-based measurements of bone formation included mineralizing perimeter (mineralizing perimeter/bone perimeter: cancellous bone perimeter covered with double plus half single label normalized to bone perimeter, %), 2) mineral apposition rate (the distance between two fluorochrome markers that comprise a double label divided by the 3-day interlabel interval, µm/d), and 3) bone formation rate (bone formation rate/bone perimeter: calculated by multiplying mineralizing perimeter by mineral apposition rate normalized to bone perimeter, μm^2^/µm/y). All bone histomorphometric data are reported using standard 2-dimensional referents and nomenclature (37). Cell-based BMAT measurements included adipocyte area fraction (adipocyte area/tissue area, %), adipocyte number (#/mm^2^) and adipocyte size (µm^2^). Adipocytes were identified as large circular, oval or ellipsoid-shaped cells bordered by a prominent cell membrane; adipocytes are easily detected because ultraviolet absorption by lipid storage droplets is negligible due to alcohol extraction of intracellular lipids during processing. These cells can be appreciated as black ‘ghosts’ surrounded by a membrane in **Supplemental Figure 1B**. The size of each adipocyte was determined by outlining the cell perimeter; the OsteoMeasure software was used to calculate cell area. On average, we counted 55 adipocytes/mouse in the ROI in distal femur metaphysis and 17 adipocytes/mouse in the ROI in distal femur diaphysis.

### Gene expression

Total RNA from BAT, WAT and tibia was isolated from 8 mice/group and individually analyzed. Whereas histomorphometry focused on a defined region of interest in femur, evaluation of gene expression included the entire tibia. Tibiae were pulverized with a mortar and pestle in liquid nitrogen and further homogenized in Trizol (Life Technologies, Grand Island, NY). BAT and WAT were directly homogenized in Trizol. Total RNA was isolated according to the manufacturer’s protocol, and mRNA was reverse transcribed into cDNA using SuperScript III First-Strand Synthesis SuperMix for qRT-PCR (Life Technologies). All quantitative polymerase chain (qPCR) reactions were done using Fast SYBR Green Master Mix (ThermoFisher), and relative quantification was determined using the ΔΔCt method.

qPCR for BAT Ucp1 gene expression was done using primers specific for mouse Ucp1 (For: GTGAAGGTCAGAATGCAAGC, Rev: AGGGCCCCCTTCATGAGGTC) and mouse 18S ribosomal RNA (Rn18s) (For: CCGCAGCTAGGAATAATGGAAT, Rev: CGAACCTCCGACTTTCGTTCT). Data represent average fold change normalized to Rn18S.

Expression levels for genes related to adipogenesis were determined for WAT and tibia using the Mouse Adipogenesis RT2 Profiler PCR Array (Qiagen). Gene expression was normalized using the averaged expression of Gapdh, Gusb, and Hsp90ab1 housekeeping genes, and relative quantification (ΔΔCt method) was determined using RT2 Profiler PCR Array Data Analysis software (Qiagen).

### Statistical analysis

#### Experiment 1

Numerical outcome variables collected in female B6 mice that were supplemented with leptin or served as controls (ad lib-fed mice and mice pair-fed to match food consumption by leptin-supplemented mice) were compared using parametric one-factor analysis of variance or nonparametric Kruskal-Wallis tests. Group mean comparisons were made using the fitted analysis of variance linear model, Welch’s two-sample t-test, or Wilcoxon-Mann-Whitney tests.

#### Experiment 2

Linear models with 5 groups (untreated B6 WT or ob/ob mice housed at 22°C, untreated B6 WT mice housed at thermoneutral temperature, and ob/ob mice receiving leptin at 0.3µg/day or a vehicle control housed at thermoneutral temperature) were used to evaluate the effects of housing temperature and leptin supplementation on BMAT.

#### Experiments 1 and 2

Residual analysis and Levene’s test were used to assess homogeneity of variance and normality. Adjustment for multiple comparisons was made by setting the maximum false discovery rate equal to 5% (38). Differences were considered significant at false discovery rate adjusted p-value ≤ 0.05. All data are presented as mean ± SD. Data analysis was performed using R version 4.1.2.

## Results

### Experiment 1: Effects of leptin supplementation on food intake, body composition, bone, and bone marrow adiposity in female B6 mice

**Figure 2:**
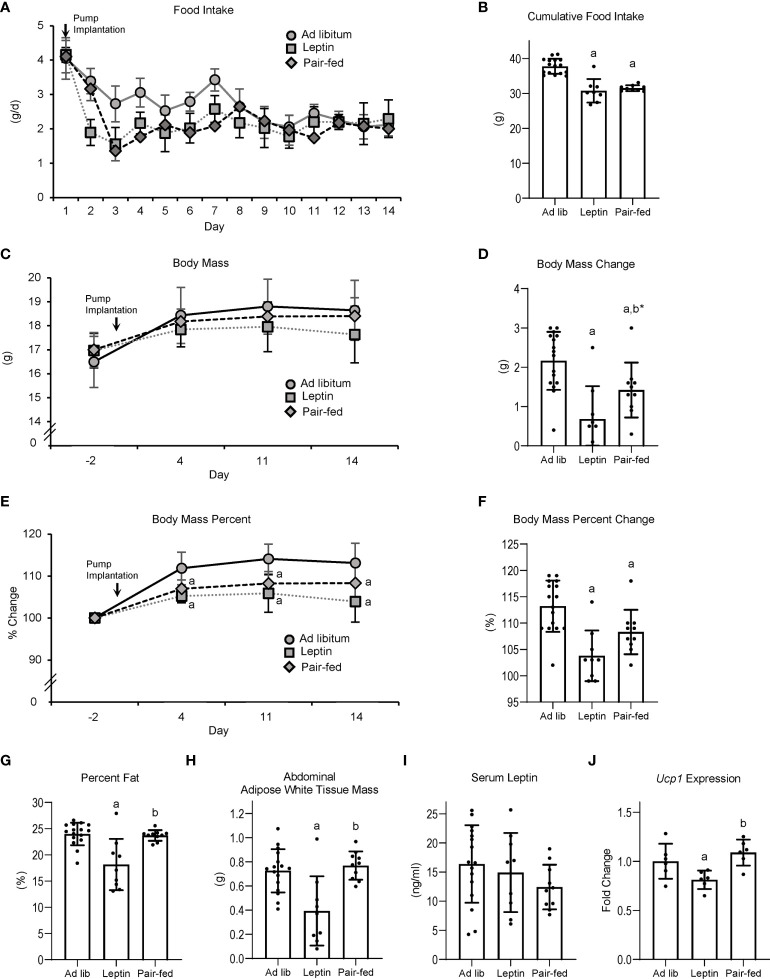
Effects of leptin supplementation and pair feeding on food intake over duration of treatment **(A)**, cumulative food intake **(B)**, body mass **(C)**, body mass change over duration of treatment **(D)**, percent change in body mass relative to baseline mass **(E)**, cumulative percent change in body mass relative to baseline mass **(F)**, percent body fat **(G)**, abdominal white adipose tissue mass **(H)**, serum leptin **(I)**, and brown adipose tissue *Ucp1* gene expression **(J)** in C57BL/6J female mice. Data are mean ± SD with individual data points shown as dots. N = 9-16/group for panels **(A–G)** and n = 8/group for panel **(H)** Analysis of variance followed by appropriate posthoc tests was used to assess differences among groups. ^a^Different from *ad lib*, FDR-adjusted P < 0.05. ^b^Different from leptin, FDR-adjusted P<0.05; ^b*^P<0.1.

The effects of leptin supplementation and pair feeding on food intake, body composition, serum leptin, and Ucp1 gene expression in BAT are shown in **Figure 2**. Food intake (panel A) decreased over time in all groups; leptin-supplemented and pair-fed mice consumed less food/day than ad lib-fed mice until day 7, after which food consumption did not differ among groups. Cumulative food intake (panel B) was lower in leptin-supplemented and pair-fed mice compared to ad lib mice. Body mass did not differ among treatment groups at any time point evaluated, including at termination of treatment (panel C). However, body mass change over the 14 days of treatment (panel D) was lower in leptin-supplemented and pair-fed mice compared to ad lib mice and tended (p = 0.06) to be lower in leptin-supplemented compared to pair-fed mice. Normalized body mass relative to baseline body mass was lower in leptin-supplemented and pair-fed mice compared to ad lib mice at all time points evaluated (panel E and F). Percent body fat (panel G) and WAT mass (panel H) were lower in leptin-supplemented mice than in ad lib or pair-fed mice. Significant differences in percent body fat or WAT mass were not detected between ad lib and pair-fed mice. Serum leptin levels (panel I) did not differ among treatment groups. Ucp1 expression in BAT (panel J) was lower in leptin-supplemented mice compared to ab lib and pair-fed mice. Significant differences in Ucp1 expression were not detected between ab lib and pair-fed mice.

**Figure 3:**
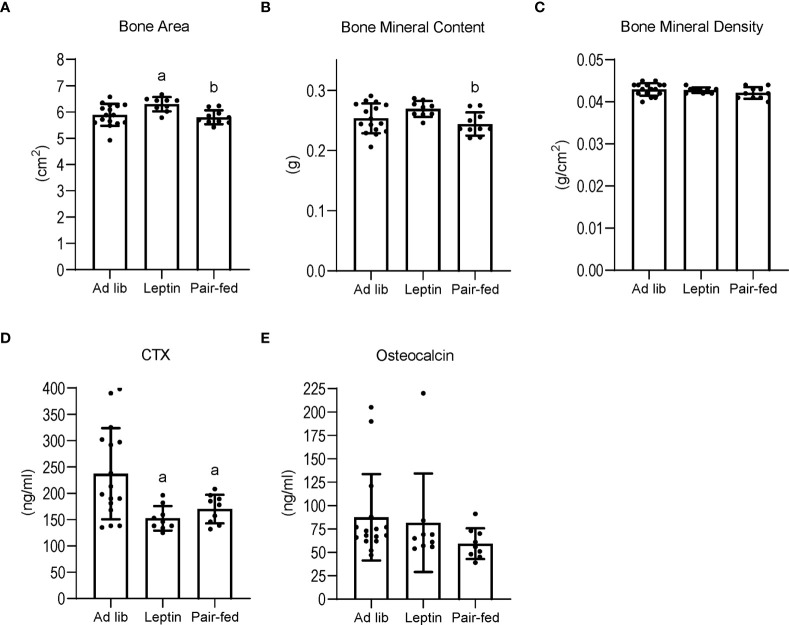
Effects of leptin supplementation and pair feeding on total body bone area **(A)**, bone mineral content **(B)**, and bone mineral density **(C)**, and on serum CTX, an index of global bone resorption **(D)** and osteocalcin, an index of global bone formation **(E)** in C57BL/6J female mice. Data are mean ± SD with individual data points shown as dots. N = 9-16/group for panels **(A–E)** Analysis of variance followed by appropriate posthoc tests was used to assess differences among groups. ^a^Different from *ad lib*, FDR-adjusted P<0.05. ^b^Different from leptin, FDR-adjusted P<0.05.

The effects of leptin supplementation and pair feeding on total body bone area, mass and density, and on serum markers of global bone turnover are shown in **Figure 3**. Bone area (panel A) was higher in leptin-supplemented mice compared to both ad lib and pair-fed mice. Bone mineral content (panel B) was also higher in leptin-supplemented mice than in pair-fed mice. Significant differences in bone area or bone mineral content were not detected between ad lib and pair-fed mice. In addition, significant differences in bone mineral density (panel C) were not detected with treatment. Serum CTX (panel D) was lower in leptin-supplemented and pair-fed mice compared to ad lib mice but leptin-supplemented mice did not differ from pair-fed mice. Serum osteocalcin levels (panel E) did not differ among groups.

**Figure 4:**
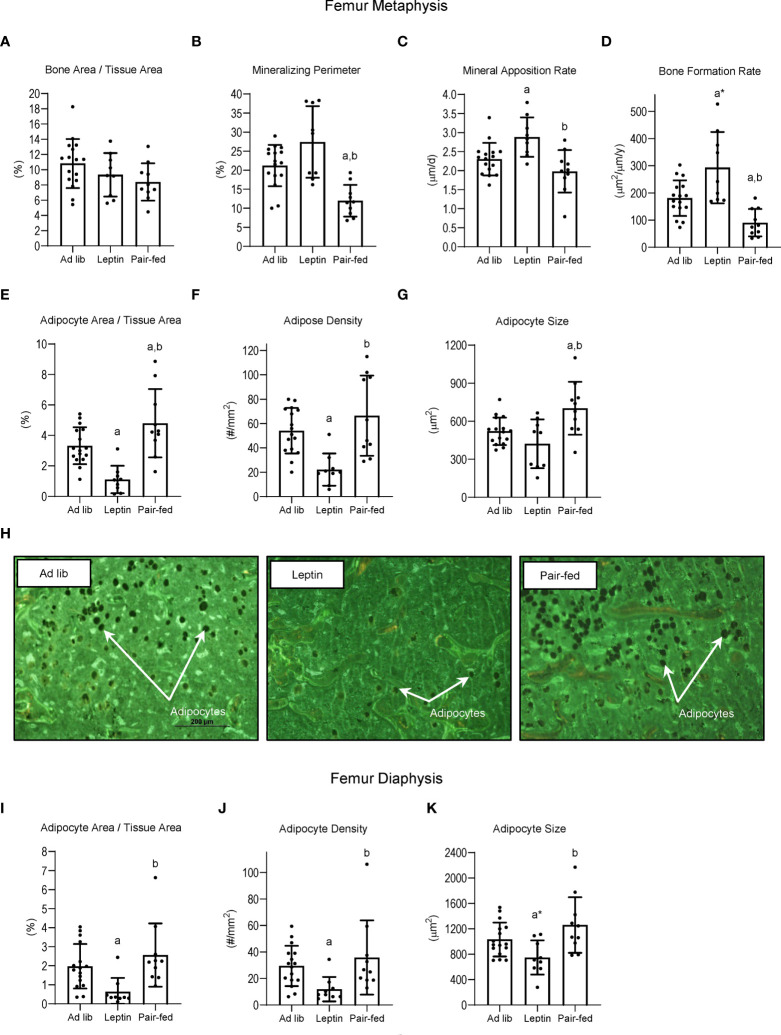
Effects of leptin supplementation and pair feeding on bone area and indices of bone formation, bone resorption, and bone marrow adiposity in distal femur metaphysis and on indices of bone marrow adiposity in distal femur diaphysis: bone area fraction (bone area/tissue area) **(A)**, mineralizing perimeter **(B)**, mineral apposition rate **(C)**, bone formation rate **(D)**, adipocyte area fraction **(E)**, adipocyte density **(F)**, and adipocyte size **(G)** in distal femur metaphysis and adipocyte area fraction **(I)**, adipocyte density **(J)**, and adipocyte size **(K)** in distal femur diaphysis in C57BL/6J female mice. Representative images of the bone marrow adiposity in femur metaphysis in each treatment group are shown in panel **(H)** Data are mean ± SD with individual data points shown as dots. N = 9-16/group. Analysis of variance followed by appropriate posthoc tests was used to assess differences among groups. ^a^Different from *ad lib*, FDR-adjusted P < 0.05; ^a*^P < 0.1. ^b^Different from leptin, FDR-adjusted P < 0.05.

The effects of leptin supplementation and pair feeding on bone, bone formation and marrow adiposity in distal femur metaphysis and on marrow adiposity in distal femur diaphysis are shown in **Figure 4**. Significant differences in bone area/tissue area in the distal femur metaphysis (panel A) were not detected with treatment. However, mineralizing perimeter (panel B) was lower in pair-fed mice compared to ad lib mice and leptin-supplemented mice, and mineral apposition rate (panel C) was higher in leptin-supplemented mice than in ad lib mice and pair-fed mice. Bone formation rate (panel D) tended (p = 0.053) to be higher in leptin-supplemented mice compared to ad lib and was higher compared to pair-fed mice. Bone formation rate was lower in pair-fed mice compared to ad lib mice. Adipocyte area fraction (panel E) and adipocyte density (panel F) were lower in leptin-supplemented mice compared to ad lib and pair-fed mice whereas adipocyte size (panel G) did not differ between ad lib and leptin-supplemented mice but was higher in pair-fed mice compared to both ad lib and leptin-supplemented mice. The differences in BMAT among the treatment groups in the distal femur metaphysis can be readily appreciated in panel H. Treatment effects on BMAT were likewise observed in the distal femur diaphysis. Adipocyte area fraction (panel I), density (panel J) and size (panel K) were lower or tended to be lower in leptin-supplemented mice compared to ad lib and pair-fed mice. However, differences in marrow adiposity were not detected between ad lib and pair-fed mice.

**Figure 5:**
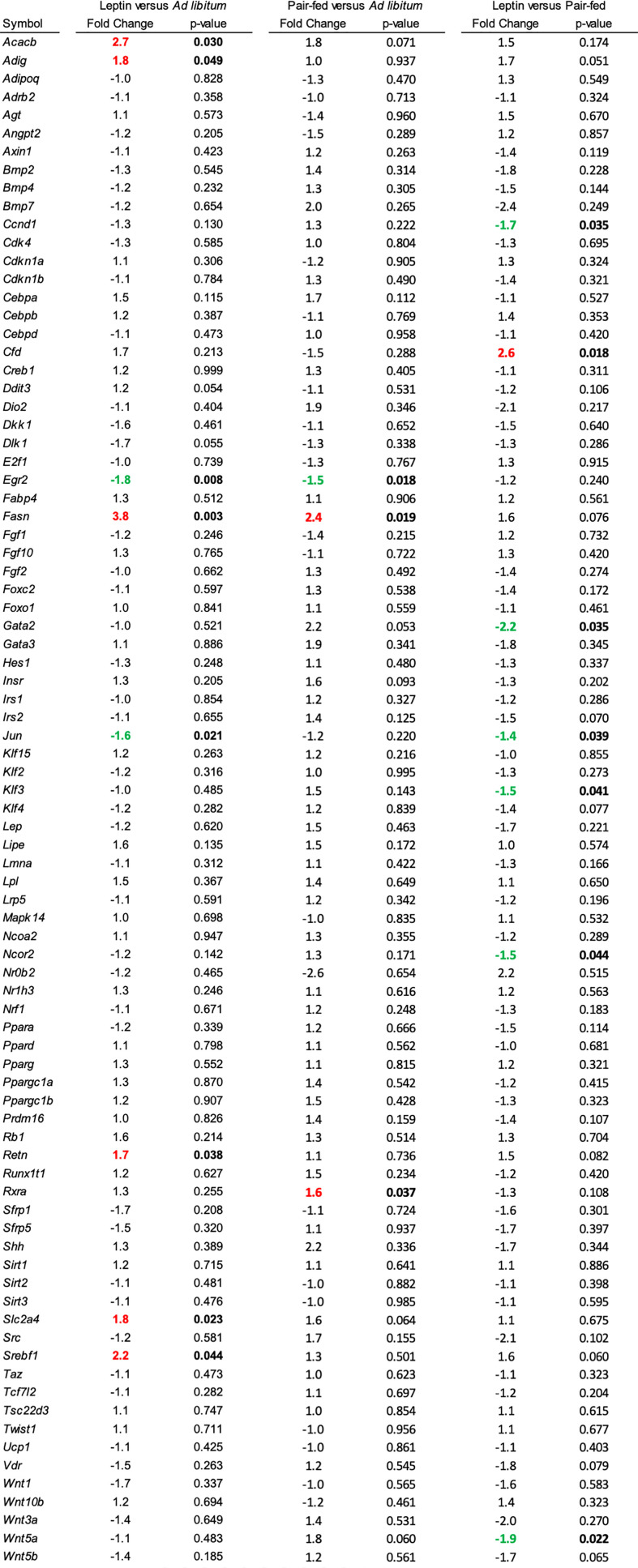
Differentially expressed genes in abdominal white adipose tissue in (1) leptin-supplemented versus *ad libitum* control mice, (2) pair-fed versus *ad libitum* control mice, and (3) leptin-supplemented versus pair-fed mice.

**Figure 6:**
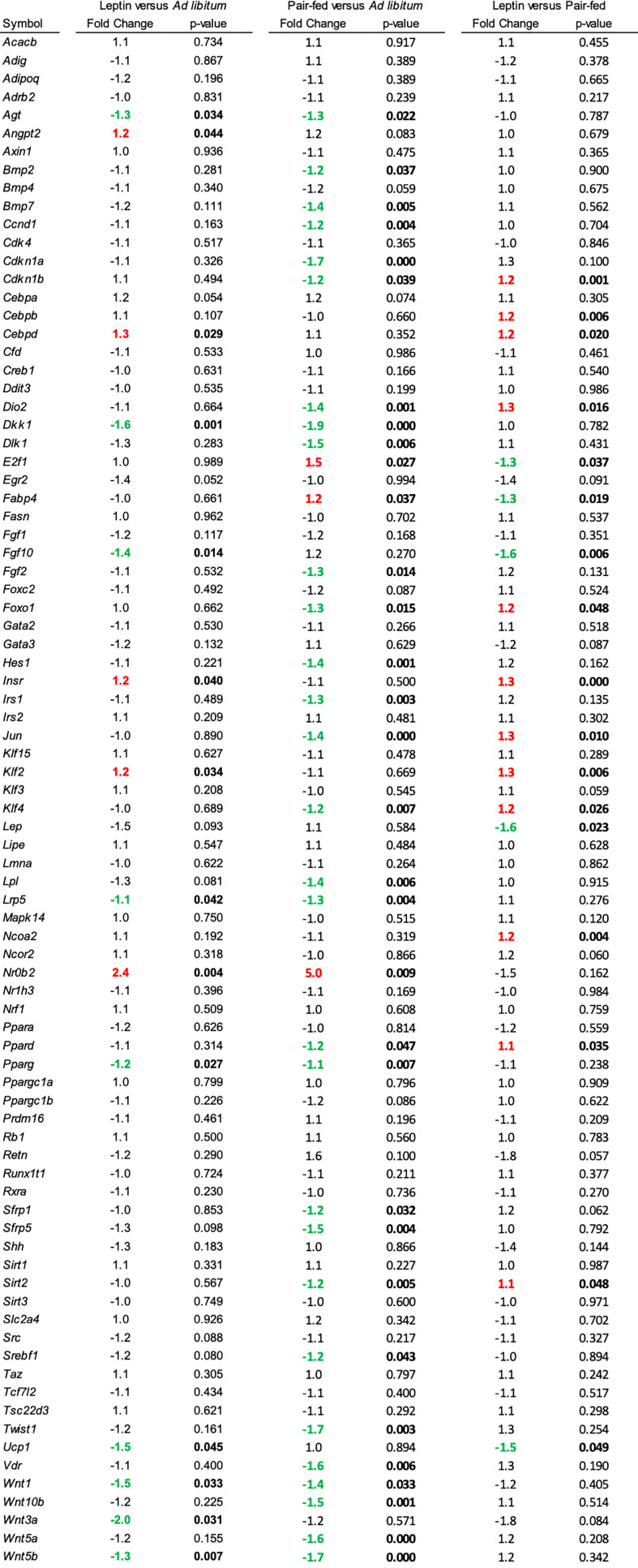
Differentially expressed genes in tibia in (1) leptin-supplemented versus ad libitum control mice, (2) pair-fed versus *ad libitum* control mice, and (3) leptin-supplemented versus pair-fed mice.

The effects of leptin supplementation on differential expression of genes related to adipogenesis in abdominal WAT and tibia are shown in **Figures 5**, **6**, respectively. In abdominal WAT, 8/84 genes were differentially expressed in leptin-supplemented mice compared to ad lib mice, 3/84 genes were differentially expressed in pair-fed mice compared to ad lib mice, and 7/84 genes were differentially expressed in leptin-supplemented mice compared to pair-fed mice. In tibia, 14/84 genes were differentially expressed in leptin-supplemented mice compared to ad lib mice, 32/84 genes were differentially expressed in pair-fed mice compared to ad lib mice, and 17/84 genes were differentially expressed in leptin-supplemented mice compared to pair-fed mice.

### Experiment 2: Effects of leptin on body composition and bone marrow adiposity in leptin-deficient ob/ob female mice

**Figure 7:**
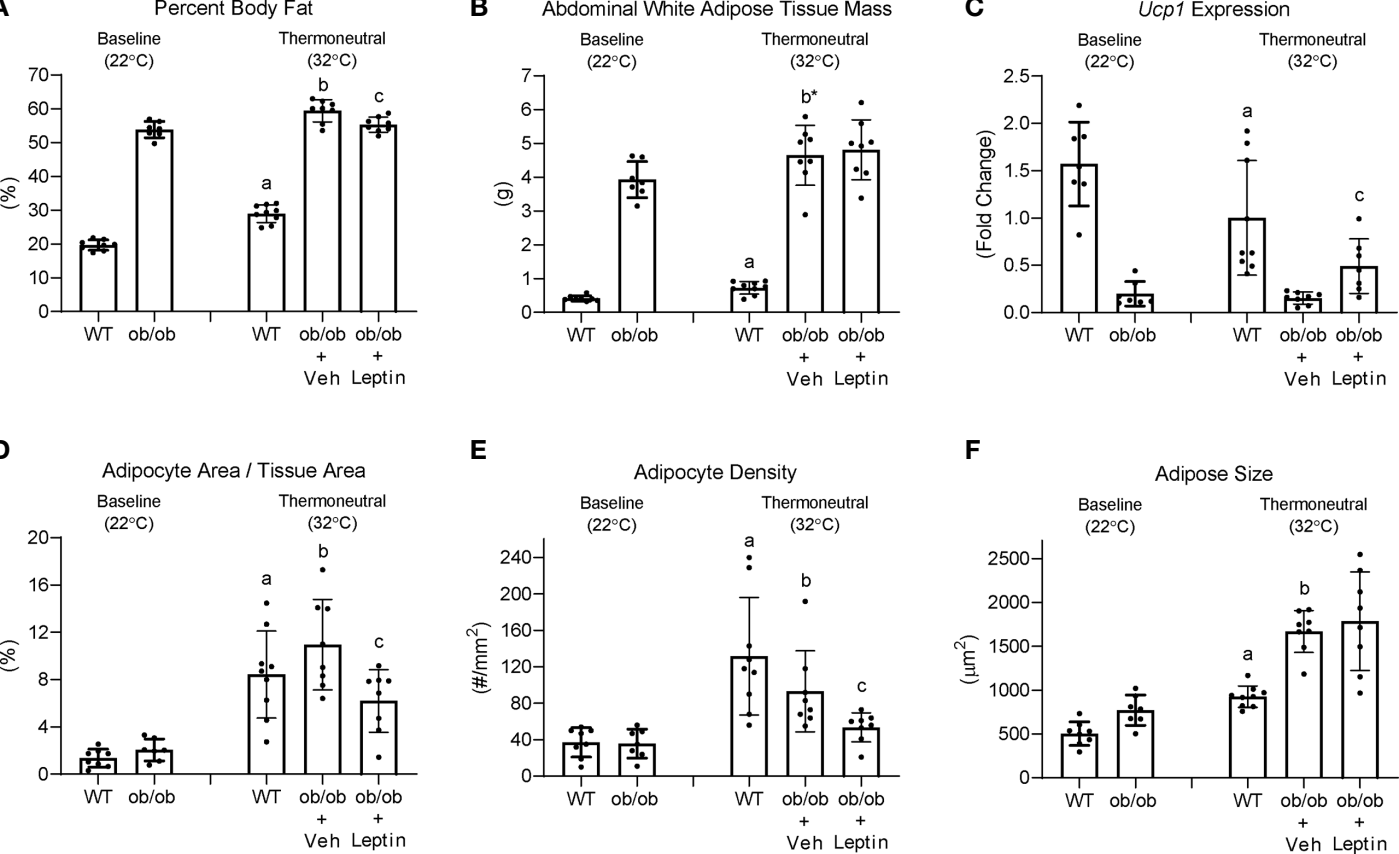
Effects of temperature and leptin administration on percent body fat **(A)**, abdominal white adipose tissue mass **(B)**, brown adipose tissue *Ucp1* gene expression **(C)** and on adipocyte area fraction **(D)**, adipocyte density **(E)**, and adipocyte size **(F)** in distal femur metaphysis in *ob/ob* female mice. Data are mean ± SD with individual data points shown as dots. N = 7-9/group. Analysis of variance followed by appropriate posthoc tests was used to assess differences among groups. ^a^WT control (32°C) different from WT baseline (22°C), FDR-adjusted P < 0.05. ^b^ *ob/ob* + vehicle control (32°C) different from *ob/ob* baseline (22°C), FDR-adjusted P<0.05; ^b*^ FDR-adjusted P < 0.1. ^c^ *ob/ob* + leptin different from *ob/ob* + vehicle control, FDR-adjusted P < 0.05.

The effects (1) of increasing housing temperature on body composition and BMAT in WT and ob/ob mice and (2) of leptin treatment on body composition and BMAT in ob/ob mice housed at 32°C are shown in **Figure 7**. Percent body fat (panel A) and abdominal WAT mass (panel B) increased in WT mice and increased (percent fat) or tended (p = 0.064) to increase (abdominal WAT) in ob/ob mice following transfer from room temperature (baseline, 22°C) to thermoneutral housing (32°C). Leptin treatment in ob/ob mice resulted in lower percent body fat but had no effect on abdominal WAT mass. As expected, Ucp1 expression in BAT (panel C) decreased in WT mice following transfer to thermoneutral housing while thermoneutral housing had no effect on Ucp1 expression in the ob/ob mice. Administration of leptin to ob/ob mice resulted in higher Ucp1 expression compared to administration of vehicle. Adipocyte area fraction (panel D), adipocyte density (panel E) and adipocyte size (panel F) increased in both WT and ob/ob mice following transfer to thermoneutral housing. Leptin treatment in ob/ob mice resulted in lower adipocyte area fraction and adipocyte density compared to vehicle. Significant differences in adipocyte size were not detected with leptin treatment in the ob/ob mice.

## Discussion

Food consumption decreased in B6 mice following transfer from room temperature to thermoneutral housing in this and prior studies (21, 22). The time course for reduced food consumption was advanced (i.e., occurred earlier) but the magnitude of the decrease was not influenced by leptin supplementation. Compared to ad lib mice, supplementation of B6 mice with leptin had no effect on serum leptin levels measured at necropsy but resulted in lower BAT Ucp1 expression, lower serum CTX, lower abdominal WAT and lower BMAT in metaphysis and diaphysis of the distal femur, and higher bone formation rate in distal femur metaphysis. Pair feeding did not closely replicate the effects of leptin supplementation on B6 mice housed at thermoneutral. Transfer of ob/ob mice from room temperature to thermoneutral resulted in higher abdominal WAT and higher BMAT in distal femur. Finally, treatment of ob/ob mice with low dose leptin reduced the increase in BMAT in distal femur following transfer to thermoneutral housing but did not impact abdominal WAT weight.

In the present study, we observed a reciprocal relationship between bone formation rate (increased) and BMAT levels (decreased) in distal femur of B6 mice following leptin supplementation. We and others noted inverse relationships between bone formation and BMAT at various skeletal sites during aging and in response to hypophysectomy, spaceflight, ionizing radiation, high fat diets and chronic heavy alcohol consumption (7–10, 14, 23, 39–42). However, as mentioned in the Introduction, a causal association between changes in BMAT levels and bone formation rate has not been demonstrated and there are numerous exceptions to this reciprocal relationship. For example, ovariectomy (ovx) in rats results in increases in osteoblast-lined bone perimeter as well as BMAT, as does increasing housing temperature of mice from room temperature to the thermoneutral range (43). Furthermore, BMAT was increased and bone formation unchanged in ovx rats following a 14-day spaceflight (39). Finally, treatment with the bone anabolic hormones fibroblast growth factor or parathyroid hormone increased bone formation in rodents without reducing BMAT (14, 44).

It remains uncertain as to whether BMAT alters the skeletal response to regulatory factors such as hormones and mechanical loads. Compared to B6 WT mice, adult ob/ob mice have higher BMAT levels. However, hindlimb unloading resulted in cancellous bone loss and changes in bone turnover in ob/ob mice indistinguishable from the skeletal response in B6 WT mice (45). Female mice housed at thermoneutral have higher BMAT and cancellous bone mass than female mice housed at room temperature but antagonizing accrual of BMAT by treatment with the nonspecific β-adrenergic receptor antagonist propranolol had minimal effect on bone mass, microarchitecture and turnover (31). Additionally, in spite of differences in BMAT, inflammation-induced bone loss was similar in distal femur metaphysis of female mice housed at room temperature and at thermoneutral (46).

The receptor tyrosine kinase cKit is required for fat storage in long bones and lumbar vertebra of mice (47, 48). Therefore, attenuation of cKit signaling provides a novel approach for investigating the regulation and function of BMAT. Results of studies performed in cKit-deficient Kit^W/Wv^ mice do not exclude the possibility that BMAT influences bone metabolism but do demonstrate that mature bone marrow adipocytes are not required for a negative bone turnover balance following ovx or during simulated microgravity (43, 49). Indeed, having normal BMAT levels appeared to attenuate bone loss in distal femur induced by simulated microgravity (49). A limitation of Kit^W/Wv^ mice is that they have hereditary macrocytic anemia, which could independently influence bone metabolism or alternatively modify the effects of BMAT (50, 51). However, adoptive transfer of purified WT (WBB6F1/J) hematopoietic stem cells into Kit^W/Wv^ mice (WT→ Kit^W/Wv^) normalized bone marrow Kit expression (52). Hindlimb unloading resulted in reduced cancellous bone in WT→ Kit^W/Wv^ mice verifying that the BMAT-deficient mice are not protected against hindlimb unloading-induced cancellous bone loss (49). Taken together, factors such as ckit signaling, mechanical loading, leptin status, sex hormones and aging clearly influence BMAT levels but strong evidence that BMAT is an obligatory regulator of bone turnover balance is largely absent. These null results do not imply that BMAT is unimportant; there is compelling evidence that BMAT plays a role in regulated hematopoiesis (1, 53–55). Also, marrow adipocytes produce hormones, adipokines and cytokines capable of positive as well as negative effects on bone metabolism (1, 56–60). In the present study, leptin and pair feeding influenced expression of genes for adipokines (Lep), hormones and growth factors (Fgf2, Fgf10, Agt, Angpt2, Bmp2, Bmp7, Dlk1), cell cycle regulators (Ccnd1, Cdkn1a, Cdkn1b), nuclear hormone receptors (Ppard, Pparg), transcription factors (Jun, Nrob2, Twist1, E2f1, Hes1, Srebf1), and signaling pathways (Sfrp1, Sfrp5, Vdr, Wnt10b, Dkk1, Wnt3a, Wnt5a, Wnt5b, Kfl2, Insr, Irs1). These changes in gene expression in bone marrow may lead to local, systemic or neuronal actions to influence bone metabolism, but definitive evidence for this is lacking.

Leptin supplementation (Experiment 1) did not result in an increase in serum leptin levels in B6 mice, when measured at necropsy. We interpret this as evidence that supplemental leptin resulted in a compensatory reduction in endogenous hormone production and an increase in leptin sensitivity. Our conclusion is supported by the observed lower abdominal WAT weight and BAT Ucp1 expression in leptin-supplemented mice compared to ad lib or pair-fed mice and lower BMAT in leptin-supplemented B6 WT as well as leptin-treated ob/ob mice. Down regulation of Lep expression in adipocytes is a potential alternative mechanism to decrease serum leptin levels and there is evidence that this mechanism is induced by hypothalamic leptin gene therapy (61). However, this alternative mechanism is unlikely because reduced abdominal WAT in the present study was not accompanied by a reduction in Lep expression. It is notable that the reduction in BMAT accrual in leptin-treated ob/ob mice was not accompanied by a parallel decrease in abdominal WAT. This suggests that leptin inhibits BMAT accrual at much lower levels than is required to inhibit WAT accrual, a conclusion supported by dose-response studies where leptin was administered to ob/ob mice (35). Indeed, administration of leptin at a dose rate of 0.3 μg/d did not increase serum leptin levels above the detection limit of 0.5 μg/ml.

Food consumption was lower in leptin-supplemented mice compared to ad lib-fed mice for several days following osmotic pump insertion, but afterwards there was no difference in food intake among treatment groups. We interpret this as further evidence that growing mice fed a normal diet are highly leptin sensitive. In support, partially leptin-deficient heterozygote ob/+ mice adapt to partial leptin deficiency by increasing adipose tissue mass until achieving leptin levels similar to B6 WT mice (62). Treatment of ob/ob mice with leptin resulted in dose-dependent decreases in appetite (35, 63). This finding contrasts with B6 mice fed a high fat diet; when treated with leptin, obese mice exhibit an attenuated response, attributable to leptin resistance (64).

Sympathetic signaling is a positive regulator of non-shivering thermogenesis and the β-adrenergic receptor antagonist propranolol blunted thermoneutral-associated increases in BMAT and WAT (65). Thus, leptin and environmental temperature may influence BMAT levels by regulating overlapping pathways. However, it is likely that the actions of leptin on BMAT differ from the adipokine’s positive central nervous system-mediated regulatory effects on non-shivering thermogenesis. It is well established that adipocytes express leptin receptors (54, 66). Additionally, treatment with leptin at a dose rate with minimal actions on energy metabolism decreased BMAT and increased bone formation in ob/ob mice (35). In contrast, mild caloric restriction by pair feeding was associated with an increase in BMAT levels. Taken together, our results strongly suggest that leptin signaling and environmental temperature independently regulate BMAT levels.

Thermoneutral housing alters many, but not all, metabolic responses (67–70). Regarding bone, compared to room temperature, thermoneutral housing prevents premature cancellous bone loss, attenuates risperidone-induced trabecular bone loss, has modest effects on cortical response to mechanical loading, and does not impact polyethylene-particle induced osteolysis (46, 71, 72). We have shown that increasing environmental temperature from room temperature to thermoneutral impacts gene expression in WAT and bone (65). In the present study, performed at thermoneutral, leptin supplementation, but not pair feeding, increased expression of genes important to fat synthesis and turnover in WAT; Acacb (Acetyl-CoA acetyltransferase), Adig (adipogenin), Retn (resistin), Slc2a4 (facilitated glucose transporter member 4, GLUT4), and Srebf1 (Sterol regulatory element-binding transcription factor 1). The findings are consistent with fat mobilization in response to leptin supplementation. Leptin supplementation and pair feeding also altered gene expression in whole tibia. However, there was no overlap in genes differentially expressed in WAT and tibia. In general agreement with treatment-associated differences in bone histomorphometry, there were differences in gene expression between leptin-treated mice and pair-fed or ad lib controls. Taken together, the results suggest that these two leptin target tissues respond differently to the hormone.

The present studies were performed in growing female mice and evaluated (1) distal femur metaphysis (histomorphometry), a skeletal site where BMAT is closely associated with cancellous bone undergoing rapid turnover, and (2) whole tibia (gene expression). The present studies do not include controls for age-related changes in BMAT that may have occurred during the 2-week intervention. However, we have previously reported higher levels of BMAT in female mice housed at thermoneutral compared to age-matched controls housed at room temperature and pilot studies revealed no change during the evaluated age range (31, 34). Our evaluation of one sex is a study limitation because there are sex differences in bone growth and BMAT accrual (21). Additionally, adipocyte number differs with skeletal site, suggesting possible differences in regulation and function. Future studies should evaluate the effects of environmental temperature and leptin on BMAT at multiple locations in skeletally mature and aging male and female mice. Furthermore, because energy balance and thermoregulation in mice differs markedly from humans it would be enlightening to investigate the response of BMAT to environmental temperatures in additional model organisms.

Our analyses were performed at 4 levels; quantitative histomorphometry, gene expression, serum biomarkers (indices of whole-body bone turnover and fat stores) and organ mass/density. Histomorphometry examined the relationships between bone turnover and BMAT at one cancellous bone site (distal femur metaphysis) in response to treatment. Evaluation of differential expression of a panel of genes related to adipogenesis was used to compare the response to leptin in WAT and whole tibia. The tibia, in addition to adipose tissue (regulated and constitutive BMAT), contains compartment-specific distributions of bone (e.g., cortical and cancellous), cartilage, hematopoietic tissue and other tissues. Differences between our skeletal site in femur and whole tibia preclude direct comparisons between these two long bones. This being said, changes identified in a long bone are generally representative of the appendicular skeleton (1, 31, 35, 43, 47, 49, 52, 62, 65). Serum biomarkers and whole organ measurements were performed as indices of macro changes in response to treatment.

In summary, increasing environmental temperature from room temperature to thermoneutral in growing female B6 and ob/ob mice has major effects on body composition, including an increase in BMAT in the distal femur metaphysis in both genotypes and an increase in leptin levels in B6 mice. Administration of leptin lowered BMAT levels in both B6 WT and leptin-deficient ob/ob mice. Based on these findings, we conclude that increases in leptin signaling in leptin-sensitive mice and environmental temperature in normal and leptin-deficient mice have independent but opposite effects on BMAT levels. Specifically, alleviation of cold stress by housing mice at thermoneutral increases BMAT compared to room temperature housing, whereas increasing leptin signaling decreases BMAT.

## Data availability statement

The original contributions presented in the study are included in the article/**Supplementary Materials**. Further inquiries can be directed to the corresponding author.

## Ethics statement

The animal study was reviewed and approved by Institutional Animal Care and Use Committee at Oregon State University.

## Author contributions

Study conceptualization, RT, UI, KP, and AB; Study execution, KP; Data collection, KN, KP, CW, DO, and RT; Data analysis, AB; Manuscript draft, RT; Figure preparation, UI; UI takes responsibility for the integrity of the data. All authors contributed to the article and approved the submitted version.

## Funding

This work was supported by National Institutes of Health (AR060913) and National Aeronautics and Space Administration (80NSSC19K0430).

## Conflict of interest

The authors declare that the research was conducted in the absence of any commercial or financial relationships that could be construed as a potential conflict of interest.

## Publisher’s note

All claims expressed in this article are solely those of the authors and do not necessarily represent those of their affiliated organizations, or those of the publisher, the editors and the reviewers. Any product that may be evaluated in this article, or claim that may be made by its manufacturer, is not guaranteed or endorsed by the publisher.
