# Supplementary material for: Leptin and environmental temperature as determinants of bone marrow adiposity in female mice
Source: Front Endocrinol (Lausanne). 2022 Oct 6;13:959743. doi: 10.3389/fendo.2022.959743 (PMC9582271; doi:10.3389/fendo.2022.959743)
Supplement: Supplementary file 1 [file DataSheet_1.pdf]

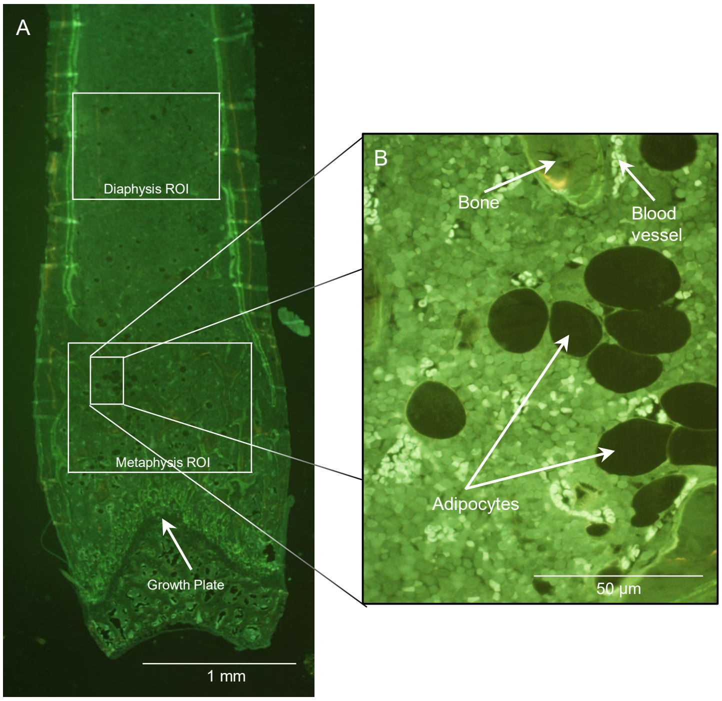

Supplemental Figure 1. Representative unstained histological section of distal femur – in a randomly chosen *ad-lib* fed-mouse) under ultraviolet illumination at 2x – depicting the regions of interest (ROI) evaluated in the distal femur metaphysis and diaphysis (panel A). The ROI in the distal femur metaphysis was located 0.25 – 1.1 mm proximal to the growth plate and averaged 0.92 mm^2 in area; any primary spongiosa and cortical bone within the ROI were excluded manually. The ROI in the distal femur diaphysis was located 2.0 – 2.7 mm proximal to the growth plate and averaged 0.63 mm^2 in area; any cortical bone in the ROI was excluded manually. Fluorochrome labels can be visualized in the low magnification image (panel A). Adipocytes can be appreciated as round, oval or elliptical-shaped black 'ghosts' surrounded by a membrane easily seen at the higher (40x) magnification (panel B).
